# Functional decline after severe COVID-19: duke activity status index for preoperative risk assessment

**DOI:** 10.3389/fmed.2025.1623423

**Published:** 2026-01-02

**Authors:** Luis Alberto Rodriguez Linares, Sarah Cerillo Lopes

**Affiliations:** 1 Department of Anesthesiology, São Paulo University, São Paulo, Brazil; 2 Department of Anesthesiology, Hospital São Luiz Rede Dor, São Paulo, Brazil; 3 Department of Anesthesiology, ABC Foundation School of Medicine (FMABC), Santo André, Brazil

**Keywords:** COVID-19, Duke Activity Status Index, functional capacity, post-COVID syndrome, anesthesia, preoperative evaluation, VO_2_ max

## Abstract

**Introduction:**

Survivors of severe COVID-19 (Coronavirus Disease 2019) frequently experience long-term functional impairment, a condition known as post-COVID syndrome. As these patients may eventually undergo surgical procedures, evaluating their functional capacity becomes essential for safe anesthetic management. The Duke Activity Status Index (DASI) is a validated, easy-to-administer tool that correlates with cardiopulmonary fitness and surgical risk.

**Objective:**

To evaluate changes in functional capacity using DASI scores in patients who survived severe COVID-19, comparing pre- and post-infection performance, and to assess implications for perioperative care.

**Methods:**

A retrospective observational study was conducted including 31 patients with a history of severe COVID-19 requiring ICU (Intensive Care Unit) admission. DASI scores were collected before and after hospitalization due to COVID-19. Associations between DASI variation and clinical variables (e.g., presence of pleural effusion, duration of intubation, ICU stay) were also explored.

**Results:**

A marked decline in functional capacity was observed after COVID-19, with lower DASI scores post-infection. The presence of pleural effusion and ultrasound abnormalities were associated with more pronounced reductions in DASI scores. A strong correlation was found between duration of intubation and length of hospital stay, and between post-COVID DASI and percentage variation.

**Conclusion:**

Severe COVID-19 has a lasting impact on functional capacity as measured by DASI. These findings highlight the need for anesthesiologists to include functional assessments in the preoperative evaluation of patients with a history of severe COVID-19. Integrating DASI into routine pre-anesthetic workup may improve risk stratification and perioperative outcomes in this vulnerable population.

## Introduction

Coronavirus Disease 2019 (COVID-19), caused by the Severe Acute Respiratory Syndrome Coronavirus 2 (SARS-CoV-2), remains a significant global health challenge. Although most individuals recover from the acute phase, a considerable subset—particularly those requiring hospitalization or intensive care unit (ICU) support—develop persistent symptoms and functional limitations, collectively known as post-COVID syndrome or long COVID (1, 2). Severe COVID-19 is frequently defined by acute hypoxemic respiratory failure and the need for mechanical ventilation, with some studies reporting intubation rates as high as 38% in hospitalized patients (3, 4).

Survivors of COVID-19-related acute respiratory distress syndrome (ARDS) often exhibit substantial long-term disability. Up to 60% report functional limitations several months after ICU discharge (5), and recent cohort studies have confirmed impaired pulmonary function and reduced exercise tolerance extending beyond 1 year (6, 7). These lingering symptoms—including fatigue, dyspnea, and cognitive dysfunction—can significantly affect patients’ ability to perform daily activities and tolerate physiological stress.

Following COVID-19 infection, some patients experience persistent cardiovascular symptoms, such as fatigue, dyspnea, and reduced exercise tolerance, which significantly impact quality of life. These symptoms are often associated with myocardial dysfunction and are more prevalent in men. Electrocardiographic evaluations have shown that this cardiac dysfunction correlates with QRS complex fragmentation and the presence of arrhythmias, including atrial fibrillation and supraventricular and ventricular extrasystoles, which may serve as indicators of subclinical cardiac impairment. These findings highlight that COVID-19 can lead to long-lasting cardiovascular consequences, even in individuals who did not require hospitalization. This emphasizes the importance of identifying and monitoring patients with persistent symptoms to understand better the extent of sequelae and their impact on cardiovascular health (8).

These persistent impairments challenge routine preoperative assessment, particularly in patients presenting for elective or high-risk surgery. Traditional risk stratification tools often overlook the effects of post-viral syndromes, necessitating more individualized approaches. Simple, validated instruments like the Duke Activity Status Index (DASI) offer a practical alternative by estimating maximal oxygen uptake (VO_2_ max), which is a key indicator of cardiorespiratory fitness and surgical risk (9–11).

Lower DASI scores have been associated with increased perioperative complications, particularly in patients with cardiovascular and pulmonary comorbidities (12, 13). Recent guidelines have emphasized the importance of functional capacity assessment in the preoperative setting, especially in populations recovering from critical illness or with uncertain physiologic reserve (14, 15).

This study aims to evaluate functional capacity using DASI scores in survivors of severe COVID-19, comparing pre- and post-infection performance. We further aim to highlight the value of incorporating functional assessments into anesthetic risk evaluation to optimize perioperative outcomes in this growing patient population.

### Justification and objective

Justification: Survivors of severe COVID-19 often present with lasting functional impairment. Preoperative assessment frequently overlooks this. Using the DASI score enables objective evaluation of cardiopulmonary capacity, improving anesthetic risk stratification in post-COVID patients. Primary objective: To assess the variation in functional capacity using the DASI score in patients who survived severe COVID-19, by comparing pre- and post-hospitalization scores, to evaluate its relevance for preoperative assessment and anesthetic planning.

## Materials and methods

### Study design and participants

We conducted a retrospective observational study at a tertiary care hospital. Eligible participants were adult patients who survived severe COVID-19, defined by the need for hospitalization with respiratory support (including orotracheal intubation and/or ICU admission). The key inclusion criterion was survival of a documented episode of severe COVID-19 followed by hospital discharge. Patients of any age, sex, or comorbidity profile were included, provided they had recovered sufficiently to undergo a post-discharge functional assessment. Exclusion criteria were patient refusal to participate or death before follow-up data collection.

Between March 26, 2020 and June 10, 2021, a total of 212 patients were hospitalized with severe COVID-19 at our institution. Among these, one patient died shortly after ICU discharge before functional assessment could be performed. Of the remaining survivors (*n* = 211), 31 patients consented to participate and completed the post-discharge evaluation, yielding a final analyzed cohort of 31 patients. No formal sample size calculation was performed for this exploratory study, as we included all eligible survivors from the study period. This approach reflects the inherent difficulties in recruiting a larger homogeneous cohort of severe COVID-19 survivors, a population with high acute-phase mortality and significant loss to follow-up.

**FIGURE 1:**
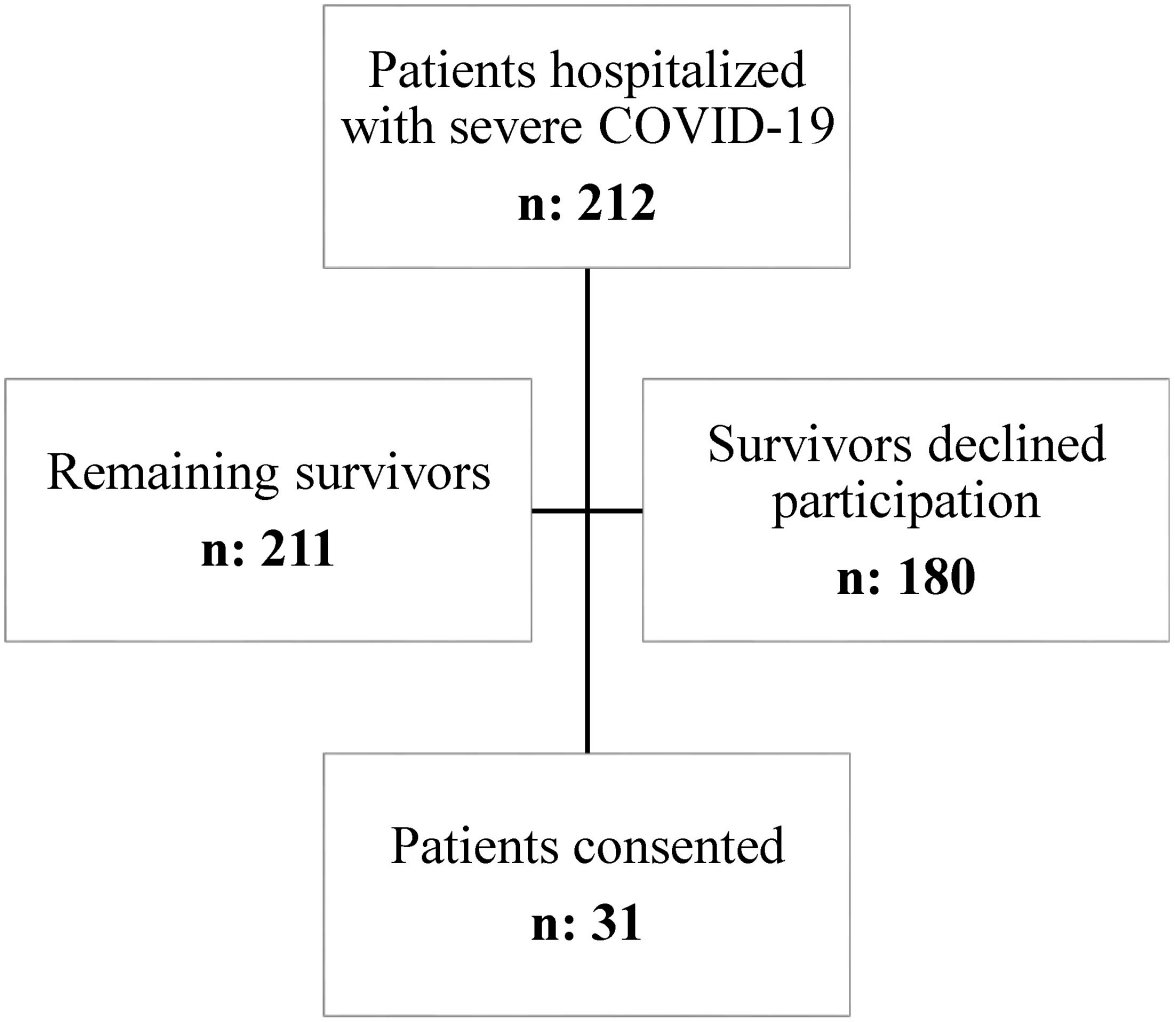
Flowchart summarizing the selection of study participants.

Flowchart summarizing the selection of study participants. A total of 212 patients with severe COVID-19 were assessed for eligibility (hospitalized during the study period), of whom 211 survived to hospital discharge. Among these survivors, 31 patients consented to participate and were included in the analysis, while 180 survivors either declined participation or were lost to follow-up. This diagram illustrates the inclusion and exclusion process, highlighting the final sample size and reasons for exclusion Figure 1.

All participants provided written informed consent prior to inclusion. The study protocol was approved by the Institutional Ethics Committee (CAAE: 37658720.4.0000.0087).

### Functional capacity assessment

The Duke Activity Status Index (DASI) questionnaire was used to evaluate functional capacity. DASI is a validated, self-administered tool that estimates functional performance based on a patient’s ability to perform daily activities. Two sets of DASI scores were collected:

1. Pre-COVID DASI scores were obtained retrospectively through patient self-report, reflecting their perceived functional capacity before COVID-19 infection as they were in in acute care setting.
2. Post-COVID DASI scores were recorded 2 months after hospital discharge, during a scheduled outpatient follow-up evaluation.

### Statistical analysis

All statistical analyses were performed using SPSS software, version 22.0 (IBM Corp., Armonk, NY, United States). Descriptive statistics were used to summarize quantitative variables (mean, median, standard deviation, interquartile range, minimum, and maximum) and qualitative variables (absolute frequencies and percentages). Normality of continuous variables was assessed using the Shapiro–Wilk test.

For comparisons between independent groups (e.g., presence of pulmonary consolidation or pleural effusion), either Student’s *t*-test or Mann–Whitney *U* test was applied, depending on the distribution of the data. Paired comparisons between pre- and post-COVID DASI scores were evaluated using the Wilcoxon signed-rank test. Correlations between continuous variables were analyzed using Spearman’s rank correlation coefficient. A two-tailed *p*- < 0.05 was considered statistically significant.

To reinforce the robustness of our findings, additional statistical analyses were conducted.

## Results

The study population (*n* = 31) was evenly distributed by sex (48.4% male; 51.6% female). Common comorbidities included hypertension (32.3%), diabetes mellitus (12.9%), and obesity (9.7%). All patients presented with pulmonary consolidation and abnormalities on lung ultrasound that were detected before the severe COVID-19 disease. Pleural engagement was present in 64.5% of cases, and pleural effusion in 19.4%. Additionally, 32.3% had at least one ultrasound alteration. Post-COVID sequelae were highly prevalent, with dyspnea in 93.5% of patients, fatigue in 77.4%, and cognitive complaints in 80.6% (Table 1).

**TABLE 1:** Qualitative variables.

Variable	n (%)
Sex—Male	15 (48.4%)
Sex—Female	16 (51.6%)
Hypertension	10 (32.3%)
Diabetes mellitus	4 (12.9%)
Obesity	3 (9.7%)
Pulmonary consolidation	31 (100%)
Pleural engagement	20 (64.5%)
Pleural effusion	6 (19.4%)
Post-COVID dyspnea	29 (93.5%)
Fatigue	24 (77.4%)
Cognitive complaints	25 (80.6%)

**FIGURE 2:**
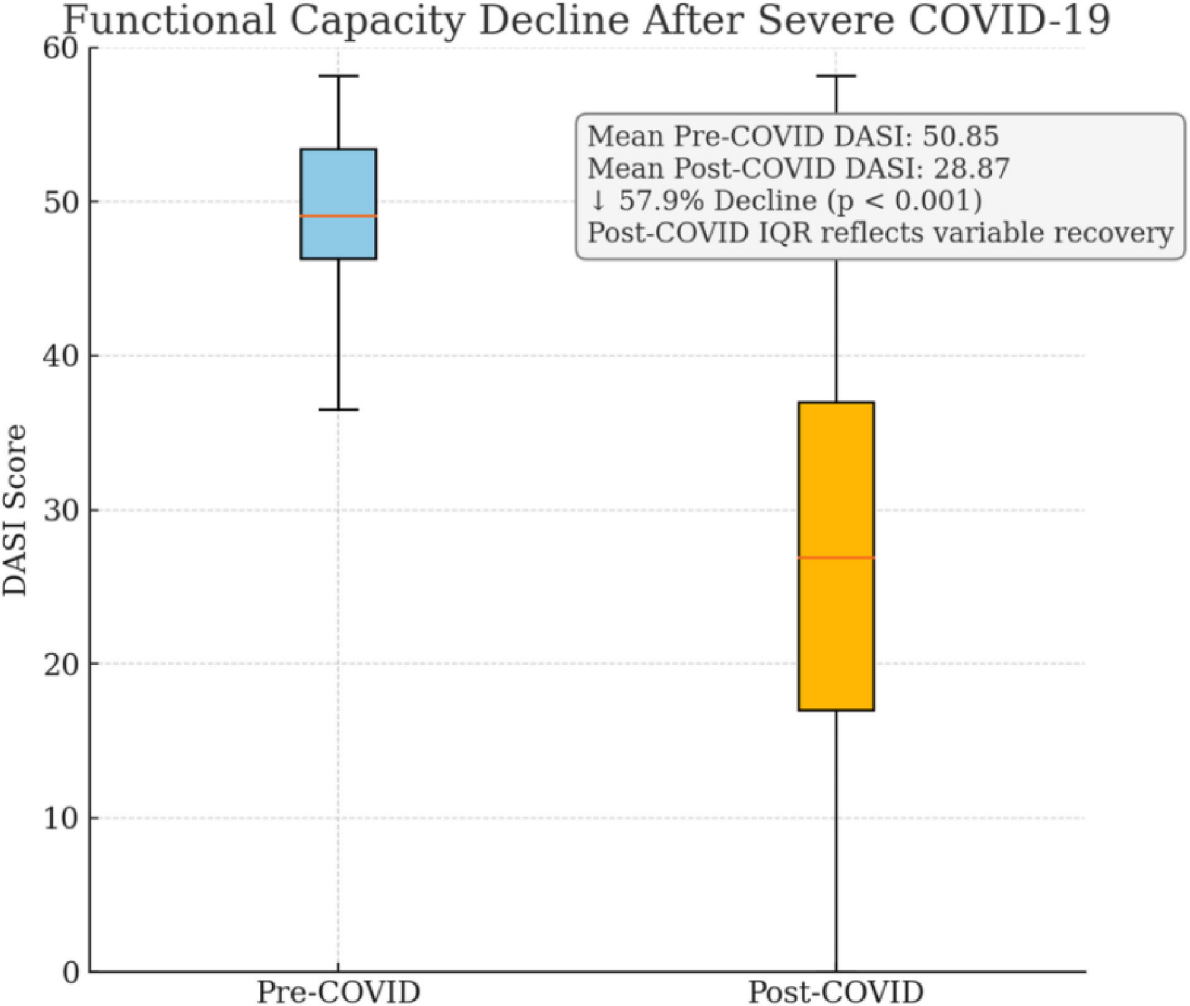
Comparison of Duke Activity Status Index (DASI) scores in survivors of severe COVID-19 before and after infection. Mean DASI scores declined from 50.85 to 28.87, representing a 57.9% reduction (*p* < 0.001). The post-COVID interquartile range reflects considerable heterogeneity in recovery, highlighting the need for individualized perioperative evaluation.

The average hospital stay among the 31 patients was 18.0 ± 10.22 days, with a median of 20 days and a wide range from 0 to 40 days, reflecting significant variation in clinical severity. The mean duration of orotracheal intubation was 12.7 ± 6.76 days, with durations ranging from 0 to 25 days, consistent with prolonged ventilatory support typically observed in severe COVID-19 cases. Regarding functional capacity, the Duke Activity Status Index (DASI) demonstrated a statistically significant decline following COVID-19. The pre-COVID DASI score averaged 50.85 ± 7.5, with a narrow interquartile range (IQR = 12.0), indicating relatively good baseline functional capacity. Post-COVID, the mean DASI score dropped to 28.87 ± 16.98 (IQR = 32.5), a highly significant reduction (*p* < 0.001, Wilcoxon test). This decline is further quantified by the DASI Variation (%), which averaged 57.93% ± 30.27, confirming that functional impairment persisted in the majority of patients (Figure 2).

The decline in DASI scores post-COVID was statistically significant (*p* < 0.001), with a very large effect size (Cohen’s *d* = 8.17), indicating a clinically meaningful reduction in functional capacity. The 95% confidence interval for the DASI score difference ranged from 21.00 to 33.75, further supporting the magnitude and precision of the observed decline. Importantly, the DASI’s responsiveness makes it a suitable choice in studies with sample-size constraints, as shown in perioperative recovery cohorts (3), pulmonary rehabilitation programs in post-COVID patients (6), and even small interventional trials (7). Its ability to capture subtle yet clinically relevant declines in functional status reinforces the validity of our observations, even in a limited cohort. These considerations mitigate concerns regarding statistical power and underline the appropriateness of DASI for this analysis.

We also performed an exploratory multivariate linear regression to adjust for potential confounders including age, sex, number of comorbidities, and duration of orotracheal intubation. The model did not reach statistical significance (*R*^2^ = 0.086; *p* = 0.658), and none of the individual predictors showed significant associations with the degree of DASI decline (*p* > 0.05). These results suggest that the observed functional decline was relatively consistent across clinical and demographic subgroups in this cohort.

In addition, a logistic regression was conducted to assess predictors of severe functional decline, defined as a DASI score reduction greater than the median. The model incorporated age, sex, comorbidities, hospital stay, intubation duration, and presence of pleural effusion. Although none of the variables reached statistical significance (*p* > 0.05), age demonstrated a borderline association (*p* = 0.088). The model’s limited explanatory power (Pseudo *R*^2^ = 0.1253) suggests that the likelihood of severe functional decline was broadly distributed across subgroups, not clearly driven by the included covariates.

Together, these analyses underscore the substantial and widespread impact of severe COVID-19 on functional capacity, regardless of demographic or clinical baseline characteristics.

The statistically significant differences between pre- and post-COVID DASI scores underscore the substantial impact of severe COVID-19 on cardiopulmonary functional reserve, an essential consideration for preoperative risk stratification and anesthetic planning, particularly in patients undergoing elective or high-risk procedures (Table 2).

**TABLE 2:** Suggested integration of DASI scores into clinical anesthesia workflows, including ASA classification, ERAS (Enhanced Recovery After Surgery) optimization, and surgical decision-making.

Clinical application	Role of DASI	Suggested action based on DASI score
ASA physical status classification	Support reclassification (e.g., ASA II vs. III) by quantifying VO_2_ max	Consider higher ASA class if DASI < 34 (VO_2_ max < 10 mL/kg/min)
Preoperative functional assessment	Provide objective measure of functional limitation in long COVID	DASI < 34 indicates poor functional status; flag for further eval
ERAS pathway optimization	Identify patients needing individualized ERAS modifications	Adapt fluid, analgesic, and mobility protocols for DASI < 34
Surgical timing decision	Assist in determining safe timing post-COVID based on recovery	Delay surgery in elective cases if DASI remains significantly reduced
Prehabilitation targeting	Target patients with low DASI scores for prehabilitation/pre-op rehab	Refer to physio/nutrition for DASI < 34 or decline > 50% from baseline

A DASI score < 34 corresponds to a VO_2_ max < 10 mL/kg/min and may indicate increased perioperative risk.

**FIGURE 3:**
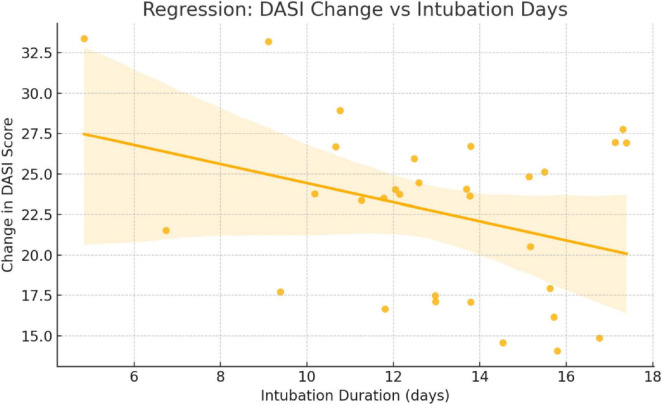
Scatter plot with regression line: association between DASI score change and intubation duration.

**FIGURE 4:**
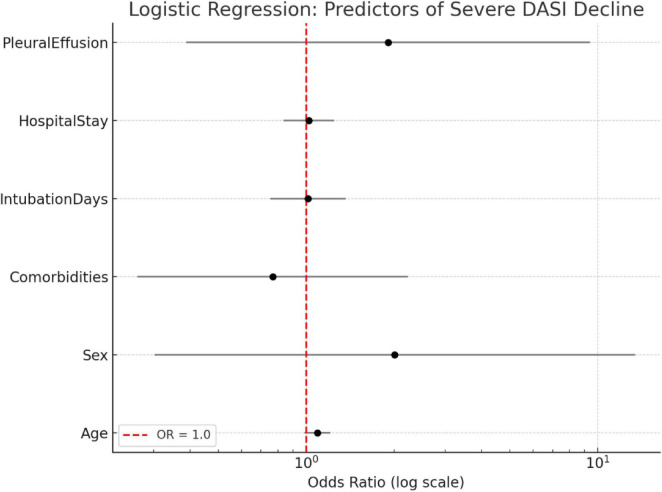
Correlation between clinical variables and DASI decline.

A strong positive correlation was observed between length of hospital stay and duration of orotracheal intubation (IOT) (ρ = 0.75, *p* < 0.001), indicating that patients requiring prolonged ventilatory support tended to have longer ICU admissions (Figure 3). Additionally, there was a powerful positive correlation between post-COVID DASI scores and DASI variation percentage (ρ = 0.90, *p* < 0.001) (Figure 4). This finding provides that patients with better functional recovery (higher post-COVID DASI) also had less proportional decline, reinforcing the clinical relevance of DASI as a sensitive marker of functional impairment (Table 3).

**TABLE 3:** DASI pre and post.

Variable	n	Mean	Median	SD	Min	Max	IQR	*p*-value
Hospital stay (days)	31	18.0	20.0	10.22	0.0	40.0	18.0	–
Intubation duration (days)	31	12.7	12.0	6.76	0.0	25.0	10.0	–
DASI Pre-COVID	31	50.85	53.0	7.5	23.45	58.2	12.0	<0.001
DASI Post-COVID	31	28.87	24.2	16.98	5.45	52.0	32.5	<0.001
DASI variation (%)	31	57.93	60.89	30.27	5.93	100.0	61.7	<0.001

No statistically significant correlations were observed between DASI scores and length of stay or IOT duration, indicating that individual functional outcomes may not be directly predicted by hospital metrics alone, and should therefore be evaluated independently using structured tools like DASI.

## Discussion

Our study demonstrates a profound and clinically significant decline in functional capacity among survivors of severe COVID-19, evidenced by an average 58% reduction in Duke Activity Status Index (DASI) scores. Before infection, the cohort showed relatively good baseline capacity (mean DASI 50.8), yet post-discharge evaluations revealed a mean of 28.9, consistent with poor exercise tolerance. Notably, a DASI score < 34 corresponds to < 4 metabolic equivalents of task (< 4 METs), an accepted threshold for poor functional status and a predictor of adverse perioperative outcomes (16, 17). Thus, many “recovered” patients, despite clinical stabilization, would be reclassified as high-risk under standard preoperative evaluation criteria.

Additional analyses reinforced the robustness of these findings. The decline was consistent across clinical subgroups, with no significant associations between DASI reduction and comorbidities, hospital length of stay, or duration of intubation. This suggests that the observed impairment is mainly attributable to the severe COVID-19 episode itself rather than pre-existing conditions. Although one exploratory analysis indicated a borderline association with age, the overall model lacked explanatory power, underscoring that functional vulnerability was widely distributed in this cohort.

These findings mirror international data showing that survivors of critical COVID-19 frequently develop persistent symptoms and impaired exercise tolerance collectively described as long COVID that extends well beyond the acute phase (18). Patients may appear clinically recovered at rest but remain physiologically frail, with diminished cardiopulmonary reserve. This functional deficit has direct perioperative implications: large multicenter studies confirm that postoperative pulmonary complications and mortality remain elevated for at least 7 weeks after infection, particularly when symptoms persist at the time of surgery (19, 20). In line with this, both European and American guidance recommend delaying elective surgery whenever possible and, when urgency precludes delay, performing individualized risk assessments that include objective functional evaluation (21–24).

Emerging evidence also indicates that COVID-19 vaccination may attenuate the persistence of post-COVID symptoms. Recent data suggest that vaccinated individuals, particularly those vaccinated after infection, experience lower frequencies of headache, arthralgia, and blood-pressure dysregulation compared with unvaccinated patients. Although vaccination does not abolish long COVID, these findings suggest a reduction in the severity and duration of selected sequelae (25). This context is relevant to our cohort, in whom vaccination status and type were unknown.

Beyond pulmonary sequelae, survivors of COVID-related ARDS also report impaired quality of life and high rates of psychological morbidity, including post-traumatic stress disorder, up to 1 year after discharge (26). Together, these observations underscore the multidimensional vulnerability of this population and the need for comprehensive, structured preoperative assessments.

In this context, the DASI questionnaire is highly valuable: it is validated, quick to administer, sensitive to moderate impairments, and strongly correlated with VO_2_ max and perioperative outcomes (27). Unlike reliance solely on comorbidities or subjective appraisal, DASI provides an objective measure of functional reserve. In our cohort, the nearly 60% decline translated into tangible limitations e.g., transitioning from easily climbing several flights of stairs pre-COVID to struggling with a single flight post-COVID. Such deficits have immediate implications for anesthetic planning (ASA Physical Status classification) and surgical timing.

Routine incorporation of DASI into the pre-anesthetic evaluation may therefore enhance perioperative safety. By identifying “hidden” impairments, anesthesiologists can tailor management strategies, including lung-protective ventilation, regional anesthesia techniques, and referrals for prehabilitation or pulmonary rehabilitation (16, 27–30). Moreover, structured use of DASI can support decisions to postpone elective procedures until functional recovery is adequate, potentially reducing perioperative morbidity (16, 31–33).

In summary, severe COVID-19 results in substantial and lasting reductions in functional capacity even in patients who appear clinically recovered. The DASI captured this decline and offers a pragmatic, evidence-based method for risk stratification. We strongly advocate that functional status measured objectively with tools such as DASI be systematically interrogated during the preoperative evaluation of COVID-19 survivors to ensure timely identification of high-risk patients and individualized perioperative care.

### Study limitations

Our sample size (*n* = 31) was modest, which may limit generalizability. This reflects the inherent difficulty of recruiting homogeneous cohorts of severe COVID-19 survivors given high acute-phase mortality and substantial attrition. We deliberately selected the DASI for its validated sensitivity to detect moderate changes in capacity, feasibility in small-sample/retrospective settings, and strong correlation with VO_2_ max and perioperative outcomes (16, 31–33). Comparable studies in ICU survivors and post-COVID cohorts have successfully used DASI in modest samples to capture clinically meaningful differences (34–37), supporting its methodological robustness. Pre-COVID DASI scores were retrospectively self-reported (possible recall bias), yet the magnitude and consistency of the observed decline, together with convergent evidence from international cohorts, support the validity of our findings. Finally, vaccination status and vaccine type were unknown, precluding adjustment for potential vaccine effects on long-COVID manifestations.

### Strengths of the study

We demonstrate a clear and persistent reduction in functional capacity after severe COVID-19, quantified by an average 58% decrease in DASI scores. Even after apparent clinical recovery, many patients exhibited low exercise tolerance, increasing perioperative risk. DASI proved practical and sensitive for identifying these limitations and for supporting individualized perioperative planning. The impairment was widespread and not solely dependent on comorbidities or hospitalization duration, emphasizing the need for individualized evaluation.

### Future directions

Larger multicenter cohorts are needed to validate the prognostic value of preoperative DASI in post-COVID surgical populations. Prospective studies should determine whether DASI-guided pathways (e.g., targeted prehabilitation, altered anesthetic strategies, delayed elective surgery) improve postoperative outcomes. Direct comparisons of DASI with CPET or measured METs could clarify predictive accuracy and cost-effectiveness in routine practice (38). Integrating DASI with frailty measures and CPET (when available) may yield a comprehensive strategy for preoperative risk stratification, aligning anesthetic practice with evolving recommendations (39). Ultimately, embedding functional capacity assessment into perioperative workflows offers a pragmatic, evidence-based approach for the growing population of COVID-19 survivors (30, 32, 38–40).

## Conclusion

In this study, we identified a marked and sustained reduction in functional capacity among survivors of severe COVID-19, as measured by the Duke Activity Status Index. Despite apparent clinical recovery, patients exhibited significant cardiopulmonary limitations, underscoring the long-term physiological burden of critical illness.

These findings have clear perioperative implications. Functional capacity remains a central determinant of anesthetic risk, and our results highlight the value of incorporating simple, validated tools such as the DASI into routine preoperative assessment. Objective evaluation enables early recognition of vulnerable patients, supports individualized anesthetic planning, and may contribute to reducing perioperative complications in this high-risk population.

As the number of surgical candidates with post-COVID sequelae continues to rise, anesthesiologists should adopt a function-based approach to risk stratification rather than relying solely on traditional comorbidity profiles. Future multicenter studies are warranted to validate these observations and to establish the prognostic role of DASI in predicting perioperative outcomes in post-COVID patients.

## Data Availability

The original contributions presented in this study are included in this article/supplementary material, further inquiries can be directed to the corresponding author.
